# Loperamide Induced Torsades de Pointes: A Case Report and Review of the Literature

**DOI:** 10.1155/2016/4061980

**Published:** 2016-02-18

**Authors:** O. Mukarram, Y. Hindi, G. Catalasan, J. Ward

**Affiliations:** ^1^Department of Internal Medicine, Texas Tech University Health Sciences Center, Permian Basin, USA; ^2^Department of Critical Care, Texas Tech University Health Sciences Center, Midland Memorial Hospital, Midland, TX, USA; ^3^Department of Clinical Pharmacology, Medical Center Hospital, Odessa, TX, USA

## Abstract

Abuse of over the counter drugs often gets overlooked by health care providers. Loperamide is one such over the counter drug that is often abused by drug addicts. We present here a case of a young male attaining euphoria from taking massive doses of loperamide. He developed Torsades de Pointes and subsequent cardiac arrest. We found similarities in the progression of myocardial electrical conduction abnormalities among loperamide and other previously known arrhythmogenic drugs. We intend to raise concern over the ease of availability of such drugs over the counter and increase the index of suspicion for over the counter drug abuse from our experience.

## 1. Introduction

Loperamide is an antimotility opioid agonist deemed safe owing to its *μ* receptor specificity and low abuse potential. It inhibits intestinal peristalsis and decreases fluid and electrolyte loss making a potent antidiarrheal agent commonly available over the counter. Adverse effects like constipation, nausea, and cramping are reported in less than 5% patients taking the prescribed dose of loperamide [1]. Previously cardiovascular side effects of loperamide were only reported as overdose cases in children [2]. However, recent literature has reported cases of cardiotoxic side effects in adults using high doses of loperamide to attain a euphoric state.

## 2. Case Presentation

**Figure 1:**
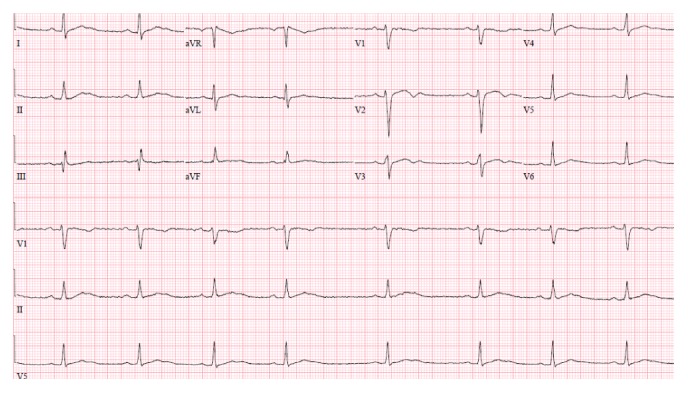
EKG on presentation showing sinus bradycardia with HR 50 bpm, PR interval 198 ms, QRS duration 122 ms, QTc interval 371 ms, and QT interval 408 ms.

**Figure 2:**
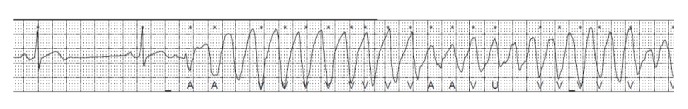
The figure shows the telemetry strip of our patient as he developed TdP.

A 26-year-old male, with history of heroin abuse, was brought to the ER after a transient episode of loss of consciousness. His vital signs upon arrival were heart rate of 50 beats/min, blood pressure of 125/60 mmHg, respiratory rate of 14 breaths/minute, and temperature of 98.9°F. He was lethargic and had symmetrical constricted pupil bilaterally, absent bowel sounds, and a normal neuromuscular, cardiac, and respiratory exam. His initial laboratory tests showed sodium of 142 mmol/L, potassium of 4.2 mmol/L, calcium of 9.1 mg/dL, and magnesium of 1.8 mg/dL; extended urine drug screen was positive only for opiates. Blood alcohol level was within normal limits. No abnormalities were noted on CT scan of the head. EKG showed sinus bradycardia with normal PR, QRS, and QT/QTc intervals (Figure 1). Soon after presentation he developed shortness of breath, diaphoresis, and sustained ventricular tachycardia with ventricular rate of 220 beats/minute. He was defibrillated 2 times with 200 J before sinus bradycardia was attained again. After defibrillation his bradycardia worsened with heart rate of 40 beats/min; however, the patient remained symptom-free. Over the course of one hour he had 2 episodes of self-limiting Torsades de Pointes (TdP) each lasting less than 10 seconds and a third ending up in pulseless ventricular tachycardia (Figure 2 shows one such episode of TdP caught on telemetric monitoring). He was defibrillated again and prompt CPR was initiated. Pulse was regained immediately and he continued to have sinus bradycardia with heart rate of 40 beats/minute. He was given 2 gm of magnesium IV and overdrive pacing was started with isoproterenol. A goal heart rate of 90 beat/minute was maintained for 48 hours. No further ventricular arrhythmias were noted; once isoproterenol was discontinued his heart rate remained between 50 and 60 beats/min. Transthoracic echocardiogram showed a normal left ventricular ejection fraction and no valvular or structural abnormalities. Cardiac angiography did not reveal any coronary pathology either. On day 3 of admission the patient admitted to taking loperamide for the past two months to relieve diarrhea associated with heroin withdrawal. Recently he had read on an internet blog that high doses of loperamide can give a similar “high” to heroin. A day prior to admission he started increasing his daily dose of the medicine to attain a euphoric state. On the day of admission his maximum ingested dose reached up to 192 mg of loperamide. At this point a serum loperamide level was sent that came back, 2 ng/mL (expected peak level after ingestion of 8 mg oral dose is 2 ng/mL or less). The patient remained hemodynamically stable and was discharged to drug rehabilitation facility on day 8 of hospitalization.

## 3. Discussion

QTc prolongation, brady-arrhythmias, and U waves have previously been reported in people abusing opioid drugs [3]. The cardiac dysrhythmia predisposing our patient to the development of QT interval prolongation and TdP was likely the persistent sinus bradycardia. Polymorphic VT has shown to occur in association with bradycardia or frequent electrical pauses [4]. Drug-induced TdP typically results from the development of early afterdepolarizations and triggered activities from prolonged repolarization. This is usually caused as a ventricular premature beat falls on a prolonged repolarization cycle. Typical feature of these TdP is an antecedent prolonged QT interval, particularly in the last sinus beat preceding the onset of the arrhythmia [5, 6]. The antecedent QTc interval in our patient was more than 500 ms prior to development of TdP. Blockade of delayed rectifying K^+^ channels on the His-Purkinje network and M cells in the heart predispose to creating heterogeneity in recovery from excitability. This creates a zone of functional refractoriness in the mid myocardial layer which forms the premise of the reentry needed to sustain TdP [7].

In rat cardiac-myocytes, opioid receptor stimulation has shown to inhibit *β*1 and *β*2 adrenergic receptor activity. This effect is a result of cross talk between signaling pathways involved. The opioid receptors are Gi/Go-coupled receptors, whereas the *β* adrenergic receptors are Gs-coupled receptors. Hence the effect of opioid receptor stimulation is to restrict the inotropic and chronotropic effect of adrenergic stimulation on the heart [8]. Researchers have found mu, kappa, and delta opioid receptors on the human heart [9]. Similar adrenergic inhibition might have a role to play in the setting of opioid overdose in human cardiac-myocytes.

Marraffa et al.'s observation showed that drug levels could not be predicted with the ingested dose and neither could the correlation of serum levels to likelihood of toxicity be made [10]. Loperamide has very poor and variable bioavailability (roughly 10 to 20%). In addition, loperamide decreases peristalsis, which can augment the systemic absorption by keeping the medication in the gastrointestinal tract for a longer period of time [1]. It is difficult to cause toxic effects while taking oral loperamide in therapeutic doses. However, as our patient took 96 tablets (192 mg), the amount systemically absorbed would be astronomical compared to the norm. The half-life elimination of loperamide is 9–14 hours; therefore, it would take 70 hours for this drug to be eliminated from the body. Prolonged consumption of the drug may lead to development of fat depots of loperamide not accounted for in the serum levels [10]. On day 3 of hospitalization, our patient still had therapeutic levels of loperamide, suggesting that this level on presentation might have been considerably higher.

Loperamide overdose has been reported repeatedly and recent literature points towards a similar pattern of cardiotoxic effects of this drug. Our case shows the electrical evolution of this arrhythmia in overdose setting and sheds light on similarities between TdP induced by loperamide and previously studied cardiotoxic drugs.

## 4. Conclusion

Abuse of over the counter drugs is often overlooked by health care providers. We intend to increase the index of suspicion of such toxicities and raise concern about the safety profile of loperamide as it still remains easily available over the counter.
